# Rethinking cervical deep lymphovenous anastomosis in Alzheimer’s disease: problems and prospects

**DOI:** 10.3389/fnagi.2026.1722759

**Published:** 2026-02-27

**Authors:** Gaiqing Wang, Haiyan Li, Sen Zhang

**Affiliations:** 1 Department of Neurology, Sanya Central Hospital (The Third People’s Hospital of Hainan Province), Sanya, Hainan, China; 2 Department of Neurology, Hainan Medical University, Haikou, Hainan, China; 3 Department of Neurology, Anyang People’s Hospital, Anyang, Henan, China

**Keywords:** Alzheimer’s disease, brain waste drainage, cerebrospinal fluid clearance, deep cervical lymphovenous anastomosis, glymphatic system, surgical translation

## Abstract

**Background:**

Deep cervical lymphovenous anastomosis (DCLVA) has been proposed as a novel surgical strategy to promote brain waste clearance in Alzheimer’s disease (AD), inspired by advances in glymphatic and meningeal lymphatic research. Early reports suggested possible cognitive benefits, yet the scientific basis of this approach remains controversial.

**Discussion:**

This Perspective critically examines the mechanistic rationale, anatomical limitations, and methodological shortcomings underlying DCLVA. The pressure disparity between cervical lymphatic and venous systems challenges the physiological feasibility of the procedure, while existing studies lack randomized design, biomarker validation, and control for anesthesia-related confounding. Ethical and translational considerations further underscore the need for rigorous preclinical and clinical evaluation before any clinical adoption.

**Summary:**

While DCLVA reflects an innovative attempt to translate lymphatic biology into surgical therapy, its current theoretical and empirical foundation is insufficient. A shift toward mechanistic validation, objective imaging biomarkers, and non-invasive modulation of lymphatic function is warranted before DCLVA can be considered a viable therapeutic option for AD.

## Introduction

Alzheimer’s disease (AD) is commonly interpreted, among several complementary hypotheses, as being partly associated with impaired clearance of metabolic waste from the brain. The discovery of meningeal lymphatic vessels (Louveau et al., 2015) and their functional integration with the glymphatic system (Da Mesquita et al., 2018) has renewed interest in brain fluid dynamics.

Building on these findings, deep cervical lymphovenous anastomosis (DCLVA) has emerged as a proposed microsurgical intervention to facilitate waste drainage. The technique is conceptually adapted from lymphovenous anastomosis (LVA), which has been extensively developed and validated in the treatment of peripheral lymphedema. In that context, LVA aims to bypass obstructed lymphatic pathways under conditions of elevated interstitial pressure and impaired lymph outflow.

However, the pathophysiological basis of AD differs fundamentally from that of peripheral lymphatic obstruction. Impaired brain waste clearance in AD primarily reflects functional disruption of glymphatic–meningeal lymphatic transport, including altered cerebrospinal fluid–interstitial fluid exchange, AQP4 depolarization, and age-related lymphatic regression, rather than mechanical lymphatic blockage. Therefore, whether surgical principles derived from peripheral LVA can be directly extrapolated to central nervous system clearance remains uncertain.

This Perspective evaluates the biological plausibility, anatomical constraints, and translational implications of applying lymphovenous bypass strategies to AD, with a focus on whether DCLVA is supported by current understanding of brain fluid dynamics and lymphatic physiology.

## Mechanistic and anatomical constraints

In peripheral lymphedema, lymphovenous anastomosis is performed under conditions of increased lymphatic pressure caused by structural obstruction, creating a favorable pressure gradient for lymph-to-venous flow. In contrast, the cervical lymphatic system operates under intrinsically low pressure, while venous pressure in the jugular system is comparatively higher.

Available physiological measurements indicate that cervical lymphatic pressure is typically below 5 cmH2O, whereas jugular venous pressure commonly exceeds 10–13 cmH2O (Wang et al., 2022). This reversed pressure gradient raises fundamental concerns regarding the feasibility of sustained lymphatic drainage following DCLVA and increases the theoretical risk of venous reflux rather than effective lymphatic outflow.

Moreover, the pronounced caliber mismatch between deep cervical lymphatic vessels and adjacent veins presents additional technical and functional challenges. Even if an anastomosis is achieved intraoperatively, long-term patency and directional flow cannot be assumed in the absence of a favorable driving force.

These biophysical disparities question the sustainability and functional efficacy of the lymphovenous connection. Even when technically successful, spontaneous collapse, thrombosis, or backflow could render the anastomosis ineffective.

**FIGURE 1:**
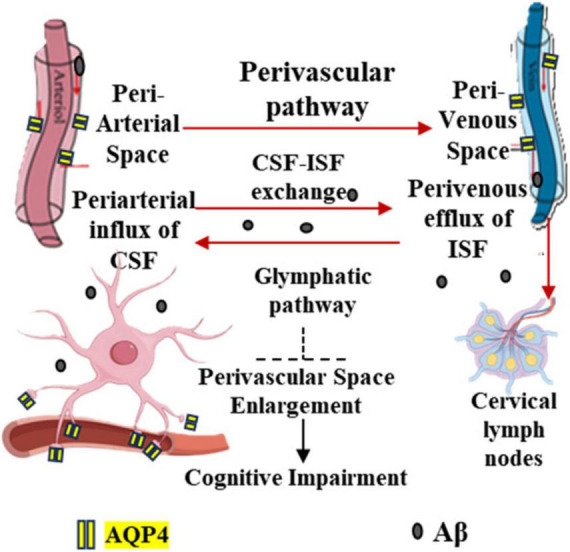
Schematic diagram of the brain glymphatic system (Aß clearance pathway).

**FIGURE 2:**
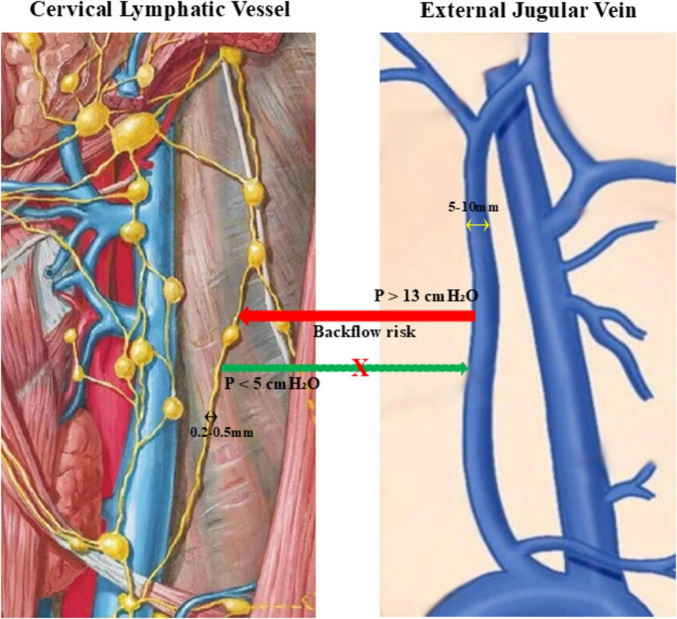
Schematic illustration of the pressure disparity between the deep cervical lymphatic vessel and the adjacent jugular vein. The diagram highlights the low-pressure lymphatic system and the comparatively higher venous pressure, demonstrating the unfavorable pressure gradient that may limit sustained lymph-to-venous drainage and increase the theoretical risk of venous reflux following DCLVA).

Figure 1 schematically illustrates the physiological glymphatic-meningeal lymphatic continuum, emphasizing the directionality of normal brain clearance, while Figure 2 depicts the adverse pressure gradient that undermines DCLVA feasibility.

## Methodological and clinical limitations

Reported clinical benefits of DCLVA are primarily based on postoperative cognitive assessments, most commonly the MMSE (Lu et al., 2022; Gan et al., 2025), without accompanying objective biomarkers of brain waste clearance, such as cerebrospinal fluid Aβ/Tau levels or imaging-based measures of glymphatic transport. In addition, existing studies lack randomized design and appropriate control groups, limiting causal interpretation. Detailed characteristics of the included clinical studies are summarized in Supplementary Table 1.

An important methodological consideration is the potential confounding effect of anesthesia. Dexmedetomidine, which is commonly used during DCLVA procedures, has been shown experimentally to enhance glymphatic transport and solute clearance by inducing sleep-like neurophysiological states. Although cognitive and behavioral assessments in published studies were conducted after apparent anesthetic recovery, none of the reports included anesthesia-matched control groups or clearance-specific biomarkers.

Therefore, while anesthesia cannot be assumed to account for postoperative cognitive changes, its potential contribution cannot be excluded under the current study designs. This limitation underscores the need for more rigorous controls and objective outcome measures in future investigations. Reported improvements rely predominantly on cognitive scales such as the MMSE (Lu et al., 2022; Gan et al., 2025), Although Chen et al measured postoperative CSF Aβ42, Aβ40, p-Tau, and total Tau and enrolled biomarker-confirmed AD patients, these assessments were not incorporated as mechanistic endpoints linked to lymphatic or glymphatic transport, nor were they accompanied by imaging-based measures of clearance dynamics (Chen et al., 2025).

Although the specific anesthetics used in DCLVA have not been explicitly reported, the potential neuroprotective effects of commonly used anesthetics such as dexmedetomidine cannot be excluded (Benveniste et al., 2017; Lian et al., 2024). Thus, postoperative improvements might reflect anesthetic effects rather than the surgery itself (Xie et al., 2013; Lee et al., 2015). None of the available studies adequately control for this confounding variable.

Given these limitations, the current clinical evidence for DCLVA remains anecdotal and inconclusive, insufficient to support any causal link between lymphovenous bypass and cognitive benefit.

## Ethical and translational considerations

Surgical interventions in neurodegenerative diseases demand strong mechanistic justification and robust safety data. Applying DCLVA to AD without such evidence risks overinterpreting preliminary findings and exposing patients to unnecessary surgical risk. Moreover, the ethical principle of “non-maleficence” requires that any invasive intervention must demonstrate clear physiological plausibility—criteria unmet in the current DCLVA literature.

Translationally, the concept of manipulating lymphatic clearance remains valuable. However, instead of invasive shunting, future strategies should explore less disruptive methods such as pharmacologic enhancement of lymphatic tone (Verghese et al., 2022), modulation of AQP4 polarization (Zhang et al., 2025), or circadian regulation of glymphatic flow (Hablitz et al., 2020). Such directions align more closely with the underlying biology of AD clearance impairment.

## Discussion

Taken together, current evidence does not support DCLVA as an effective treatment to improve brain waste clearance in AD. A key physiological barrier is the unfavorable pressure gradient between brain lymphatic vessels and cervical veins, which limits sustained drainage through DCLVA. Moreover, repeated failures of Aβ-targeting therapies highlight the complexity of AD and suggest that enhancing clearance alone may be insufficient.

As an invasive microsurgical procedure, DCLVA carries significant risks, including vascular injury, infection, lymphatic leakage, and potential damage to critical cervical neurovascular structures. Given the limited clinical experience and absence of large-scale safety data, its risk profile remains poorly defined. This uncertainty calls for cautious evaluation of risk versus benefit, careful patient selection, and rigorous monitoring in future clinical applications. Potential adverse effects must be weighed against unproven therapeutic benefits, underscoring the need for thorough preclinical and clinical investigation before broader use.

Although lymphatic dysfunction plays a role in AD, invasive surgeries like DCLVA lack strong mechanistic and biomarker validation. Enthusiasm should be tempered by these biological and clinical realities. Future research should focus on identifying reliable clearance biomarkers and developing targeted, non-invasive therapies to modulate the glymphatic-lymphatic system. Until then, DCLVA remains speculative and unsupported for clinical use.

## Data Availability

The original contributions presented in this study are included in this article/Supplementary material, further inquiries can be directed to the corresponding author.
