# Supplementary figures and images for: Rethinking cervical deep lymphovenous anastomosis in Alzheimer’s disease: problems and prospects
Source: Front Aging Neurosci. 2026 Feb 27;18:1722759. doi: 10.3389/fnagi.2026.1722759 (PMC12982382; doi:10.3389/fnagi.2026.1722759)

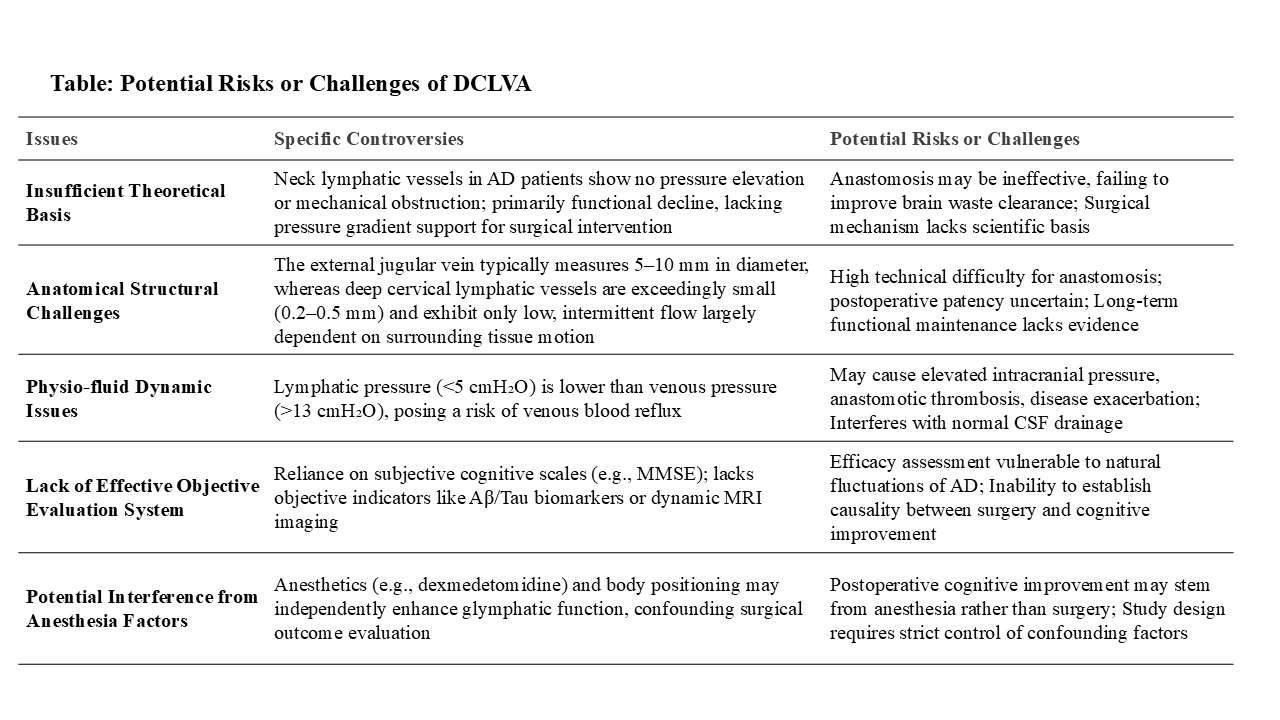

Supplement: Supplementary file 1 [file Image_1.tif]
